# Screening and enzymatic activity of high-efficiency gellan lyase producing bacteria *Pseudoalteromonas hodoensis* PE1

**DOI:** 10.1080/21655979.2019.1628882

**Published:** 2019-06-17

**Authors:** Ang Li, Hangqi Luo, Tingting Hu, Jingyu Huang, Nafee-Ul Alam, Yuan Meng, Fenbin Meng, Nartey Linda Korkor, Xiufang Hu, Ou Li

**Affiliations:** College of Life Science and Medicine, Zhejiang Sci-Tech University, Hangzhou, China

**Keywords:** Gellan, gellan-depolymerizing, gellan lyase, Pseudoalteromonas hodoensis

## Abstract

Gellan is a widely used microbial polysaccharide and one of the more effective ways to expand its application value would be to investigate the mechanism of gellan lyase and to produce gellan oligosaccharide. In this study, efficient gellan degrading bacteria were screened. One of the strains with high efficient gellan degradation capacity was labeled PE1. Through physiological and biochemical analysis of 16S rDNA, the species was identified as *Pseudoalteromonas hodoensis*. The optimum conditions for enzymatic activity and how it was affected by metal ions were determined, and the results showed that the lyase activities were much higher than those of previously reported (about 20 times). The gellan degradation products were determined by thin-layer chromatography and the oligosaccharides were determined by high-efficiency liquid chromatography to analyze the action site of lyase. This study laid a solid foundation which elucidates the production and application of gellan oligosaccharides.

**Research highlights**

● High efficiency gellan lyase producing bacteria

● Optimization of reaction conditions for gellan degradation

● Oligosaccharides were detected by TLC and HPLC to speculate the lyase action sites.

## 1. Introduction

Gellan gum is a linear acidic heteropolysaccharide produced by aerobic fermentation of *Sphingomonas elodea*. It is a microbial extracellular polysaccharide with unique physical and chemical properties as well as excellent application performance [1]. Gellan gum can be used as a new type of emulsifier, suspension agent, thickener, stabilizer, gel agent, sustained-release agent, film-forming material, and many more [2]. Gellan and its products can also be applied in the fields of food, medicine and chemical industry [3]. In order to investigate the mechanism of gellan lyase and to produce gellan oligosaccharide, would be one of the more effective ways to expand its application value [4]. Therefore, we chose to use efficient gellan lyase to improve its molecular structure and expand its application range.

Presently, two distinct reactions catalyzed by the enzyme are mainly used for the enzymatic depolymerization of polysaccharide chains: hydrolysis and lytic β-elimination(a common lysis mode of the acid polysaccharides) [5]. β-eliminative cleavage of a glycosidic bond occurs when the sugar is substituted with an acidic group next to the carbon forming a glycosidic bond which results in the breakage of the glycosidic bond adjacent to the carboxylate group, forming a new reducing end with the unsaturated sugar at the non-reducing end [6]. Because of this, the oligosaccharides of lysis products were endowed with new physiological activities. The chemical steps of this mechanism were proposed by Gacesa in 1987 [7]. According to previous studies, gellan is composed of linear tetrasaccharide repeating units of β-D-glucuronic acid, β-D-glucose,α-L-rhamnose, and β-D-glucose [8,9]. Based on the related acidic polysaccharides degradation mechanism, the gellan lyase breaks down the 1,4-glycosidic bonds associated with the β-D-glucuronic acid, forming unsaturated oligosaccharides. This resulted in reduced viscosity and molecular weight of gellan.

Currently, the industrialized degradation methods commonly used for gellan are irradiation and acid degradation, whereby the molecular weight of gellan is reduced by physical or chemical means, respectively [10]. However, these methods are in general limited by slow degradation rate, complexity and high cost of purification for the degradation products. Thereby using gellan lyase is the most ideal degradation method [3]. Furthermore, the reaction of gellan lyase can easily be monitored in real time, with the possibility of controlling the reaction and adjusting the relevant conditions during the reaction, so as to obtain the corresponding molecular weight of gellan gum for enzymatic β-elimination of oligosaccharides [11]. Gellan oligosaccharides also have antibacterial [12], antioxidant [13], and intestinal prebiotic [14] effects, which are important for the expansion of the application value of polysaccharides. However, the activity of related gellan lyase has not been strong. Therefore, this study screened for gellan lyase producing microorganisms which have a high efficient gellan degradation ability from abundant microbial resources in order to facilitate the degradation of gellan. Furthermore, we completed identification, comparative analysis and the evaluations of enzyme reaction conditions to perfect the related research of lyase.

## 2. Materials and methods

### 2.1. Screening of gellan lyase producing bacteria

In order to isolate gellan-degrading bacteria, algae samples were collected from the South China Sea. The algae had obvious gelatinous substance, so we speculated that there might be bacteria capable of degrading polysaccharides. After the algae sample was collected manually, it was immediately stored at low temperature to complete strain screening. After the 1 gram of algae sample had been thoroughly ground in the mortar, 9 ml of sterile water was used to dissolve it fully. Then, 1 ml of dissolved liquid was mixed with 9 ml deﬁned medium consisting of 0.1% (N$H_{4}$)_2_HPO_4_, 0.02% KCl, 0.01% MgSO_4_ and 0.2% gellan, with gellan as the sole carbon and energy source. Samples were then cultivated at 37°C, at a pH of 7.0 for 24 h with shaking at 240 rpm. Afterward, 100 μl from ﬂasks with registered growth were plated on Peptone-Yeast extract (PY) medium (0.2% peptone, 0.1% yeast extract) supplied with 0.5% gellan and incubated at 37°C for 5 days with gellan as the coagulant [15]. On the 5^th^ day, colonies with the visible formation of pits were isolated. Using a sterile inoculating tube, a small amount of the pits was streaked on fresh media. This was done at least three times to obtain pure single colonies for pure cultures which were further used for the study.

### 2.2. Physiological and biochemical identification of gellan-degrading isolates

The identification of the isolates was carried out by 16S rDNA sequence analysis. Firstly, DNA was extracted using a Chelex-100 genome kit. Amplification of 16S rDNA was then performed with general primers, 27F (5ʹ-AGAGTTTGATCMTGCTCAG-3ʹ) and 1492R (5ʹ-TACGGYTACCTTGTTACGACTT-3ʹ). PCR amplification was carried out with pre-denaturation at 94℃ for 3 min, 35 cycles(denaturation at 94°C,1 min; annealing at 52℃,1 min; extension at 72°C,1 min), and a final incubation at 72℃ for 5 min [16]. The purification, cloning, and sequencing of PCR products were completed at the Sangon Biotech (Shanghai) Co., Ltd. The sequencing products were listed on the Ezbiocloud website for homologous comparison [17] and the phylogenetic tree was constructed using MEGA7.0.26 software. Spore staining [18], capsule staining [19], flagellum staining [20] and gram staining [21] were carried out on high activity lyase strains and the specific morphologies of the strains were observed by scanning electron microscope [22]. Biochemical identification tubes were used to complete the tests of methyl red, Volpe test, arginine, ornithine, lysine, ONPG(O-Nitrophenyl-β-D-Galactopyranoside), sucrose, glucose, xylose, hydrogen sulfide, aesculin, urea, and nitrate to determine the physiological and biochemical properties of the bacterial strains [23].

### 2.3. Enzyme assays

Gellan lyase activity was assayed as described by Weissbach and Huritz [24]. Lyase activity was determined by increasing the absorbance continuously at 235nm using a spectrophotometer(Pye Unicam, SP8-500) based on the accumulation of unsaturated glucuronyl ends after cleaving of the glycosidic bond between glucosyl and glucuronyl residues in the molecular structure of gellan [25]. Strains were cultured in 2216 E enrichment medium and incubated at 30°C for 24 h, and then 10% of the inoculum was transferred onto PY fermentation medium for 36h. The fermented liquid was centrifuged at 8000rpm at 4°C for 15 min. The supernatant was then concentrated using a Millipore standard cassette system with an ultraﬁltration membrane (MW cut-oﬀ 10 kDa) and precooled (4°C) 50 mM Tris-HCl buffer (pH 7.0) to obtain gellan lyase. Gellan lyase (1.5 ml of crude enzyme) was added to 15 ml of 0.05% gellan solution in 50 mM Tris–HCl buﬀer, pH 7.0 and incubated at 30°C. The absorbance changes of the gellan degradation were determined by Pye Unicam Spectrophotometer at 235 nm. The active unit was defined as the activity of gellan lyase to degrade gellan oligosaccharides to produce double bonds within one hour, thus increasing the absorbance value by 0.001 [15]. This assay was used only for the determination of enzyme activity. All experiments were carried out in triplicate and the average value was generated.

### 2.4. Optimization of reaction conditions for gellan degradation

To determine the optimal conditions for gellan degradation, samples were exposed to varying temperatures and pH and various compounds (see Table 1 for details). For the pH, temperature and various compounds, the detection were in the range of 6–8, 25–40°C and 1mM of various compounds separately in 0.05% gellan solution in 50 mM Tris–HCl buffer respectively. The lyase activity was detected at 235nm by spectrophotometry every 30 min. Using the lyase activity detection method described above, the optimal conditions for gellan degradation were accordingly determined.

### 2.5. Thin layer chromatography analysis

The degrading activity and degradation products of gellan were detected and identified by thin layer chromatography (TLC) [26]. Gellan and its constituent monosaccharides were used as reference materials to compare the positions of degradation products, which were used to verify the corresponding lyase activity. 10 μl of the degradation products mixture was spotted onto a silica gel 60 F_254_ plates and developed with a solvent system consisting of *n*-butanol/acetic acid/water(5:4:1,v/v/v) in a TLC developing tank [27]. The ascending development of the TLC plate was carried out at room temperature for 2h, and then the plate was transferred to the fume hood for natural air drying. The plate was soaked in 1 l of a methanol solution containing 30%(v/v) sulfuric acid and then dried in a 110°C oven for 20 min to visualize the spots [28]. All experiments were carried out in triplicate.

### 2.6. High-performance liquid phase analysis

The lyase products were determined by HPLC to analyze the action site of lyase generally, 100 ml of the crude degradation products mixture passed through the activated carbon adsorption column. After three column volumes were washed with deionized water, 25% ethanol eluent was collected for three column volumes. Then, 25% ethanol eluent through rotary evaporation (110rpm, 55°C to evaporate the alcohol. After that, the eluent passed through the anion exchange column, collected the 50mM NaCl eluent for three column volumes. Demineralization was accomplished by dimensional exclusion chromatography (Dextran gel G-15, Scope of separation <1500Da). Glucan standards and purified products were analyzed by high-performance liquid phase (Younglin Instrument ACME-9000, Seoul, South Korea) equipped with a Sugar KS-802 column (Shodex, Tokyo, Japan) and a RI-detector at 45°C. The column heater was set at 75°C, and deionized water was used as the mobile phase at a flow rate of 1ml/min.

## 3. Results

### 3.1. Isolation of gellan lyase producing bacteria

**Figure 1.**
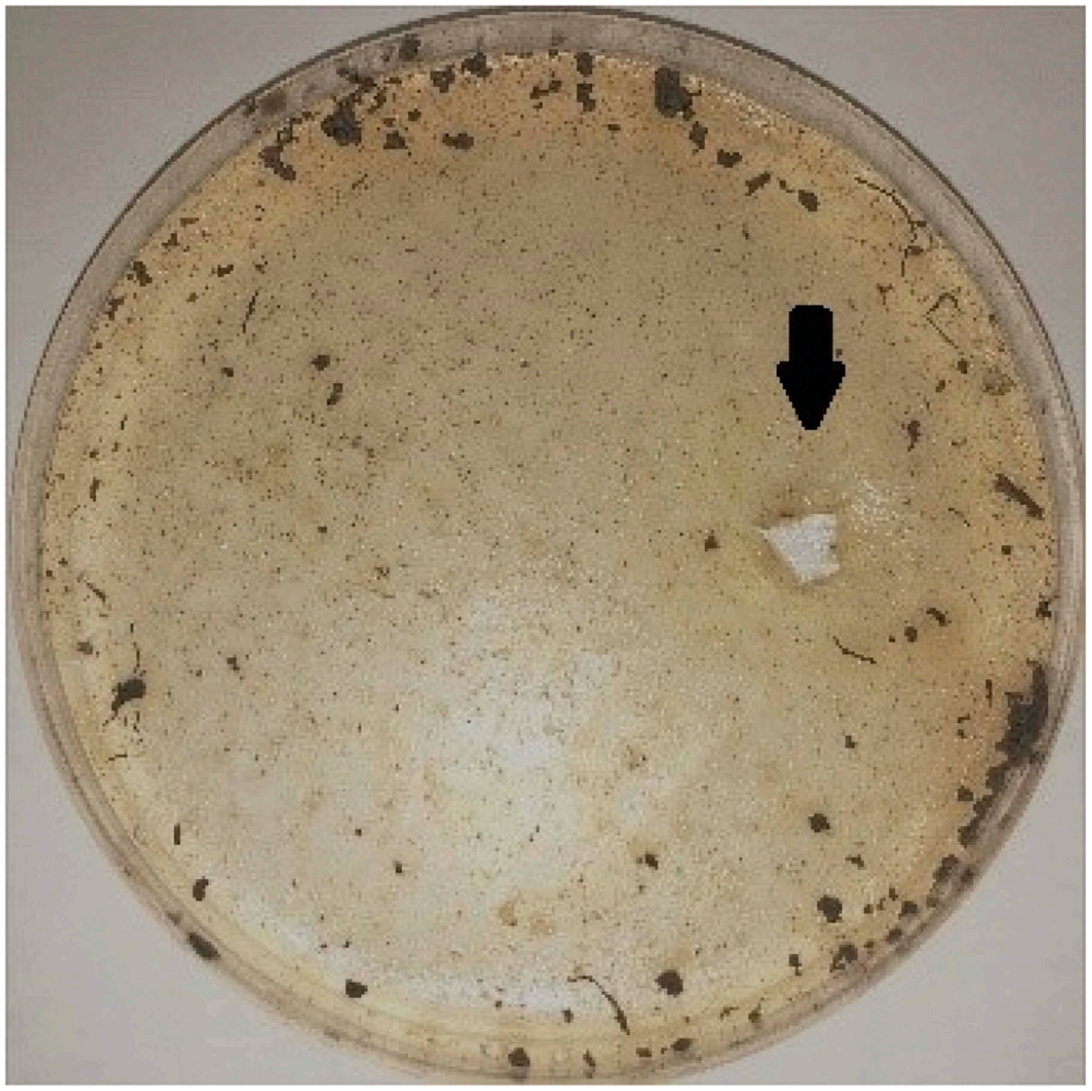
Screening plate pit. Gellan was the coagulant and the sole carbon source; as it was degraded by lyase, obvious pits would be produced on the plate. Thus, high-efficiency gellan lyase producing bacteria could be obtained by isolating and purifying the strains in the pits.

Samples were directly inoculated onto PY medium with gellan as the coagulant and the sole carbon source, and gellan lyase producing bacteria had pits forming as showed in Figure 1. Microbial growth was not easily determined because of the turbidity of the gellan samples. Using a sterile inoculating tube, a small amount of the pits was streaked on fresh media. This was done at least three times to obtain pure single colonies. The subcultures were considered pure after microscopic observation of one type of bacterium per culture. Four strains of gellan-degrading bacteria were purified from the pits of the plates, and one of them was determined to have high efficient gellan degradation capacity by preliminary lyase activity detection and was labeled PE1(NCBI registration number: MK788321).

### 3.2. Identification of gellan-degrading microorganisms

**Figure 2.**
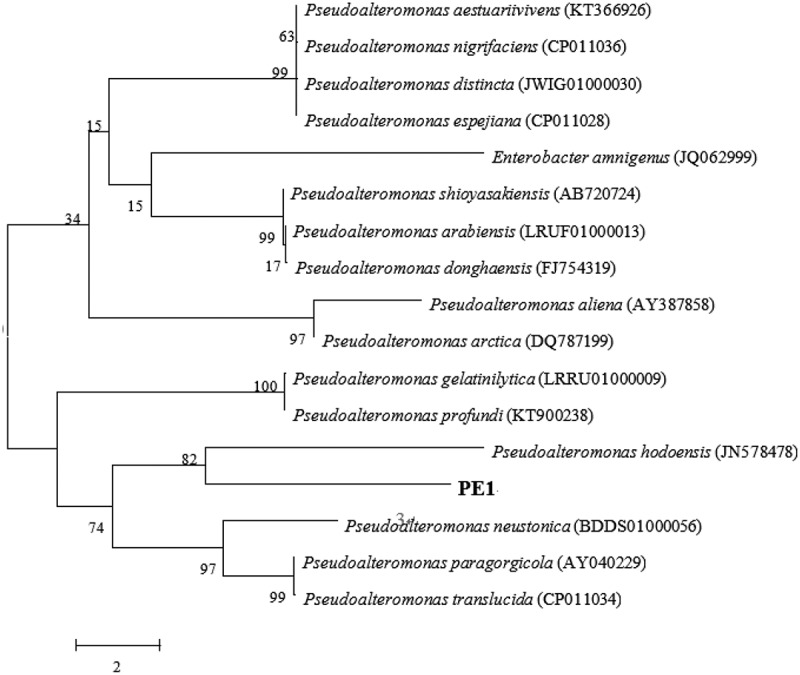
Phylogenic tree of *Pseudoalteromonas hodoensis* with related species. Neighbor-joining tree based on 16S rDNA sequences shows the position of *Pseudoalteromonas hodoensis*. The marker bar denotates the relative strain similarity.

For the identification of the gellan-degrading isolates, 16S rDNA sequencing was carried out. The PCR product amplified by conventional 16S rDNA primers was sequenced and compared with the BLAST program. Four strains of gellan lyase producing bacteria were classified (Table 2). The isolated PE1 showed high identity to *Pseudoalteromonas hodoensis* (98.95%).The phylogenetic tree was constructed to confirm the relationship with other *Pseudoalteromonas* species (Figure 2).

**Table 2.** Four strains of gellan lyase producing bacteria.

serial number	Bacteria	Similarity（%）	Completeness（%）	NCBI registration number
PE1	*Pseudoalteromonas hodoensis*	98.95%	100%	MK788321
PE2	*Achromobacter piechaudii*	99.65%	98.4%	MK909908
PE3	*Pseudomonas meliae*	98.23%	86.0%	MK909909
PE4	*Enterobacter bugandensis*	99.73%	99%	MK909914

16S rDNA sequencing was carried out. The PCR products were sequenced and compared with the BLAST program to determine the classification status of strains.

### 3.3. Morphology and physiology

**Figure 3.**
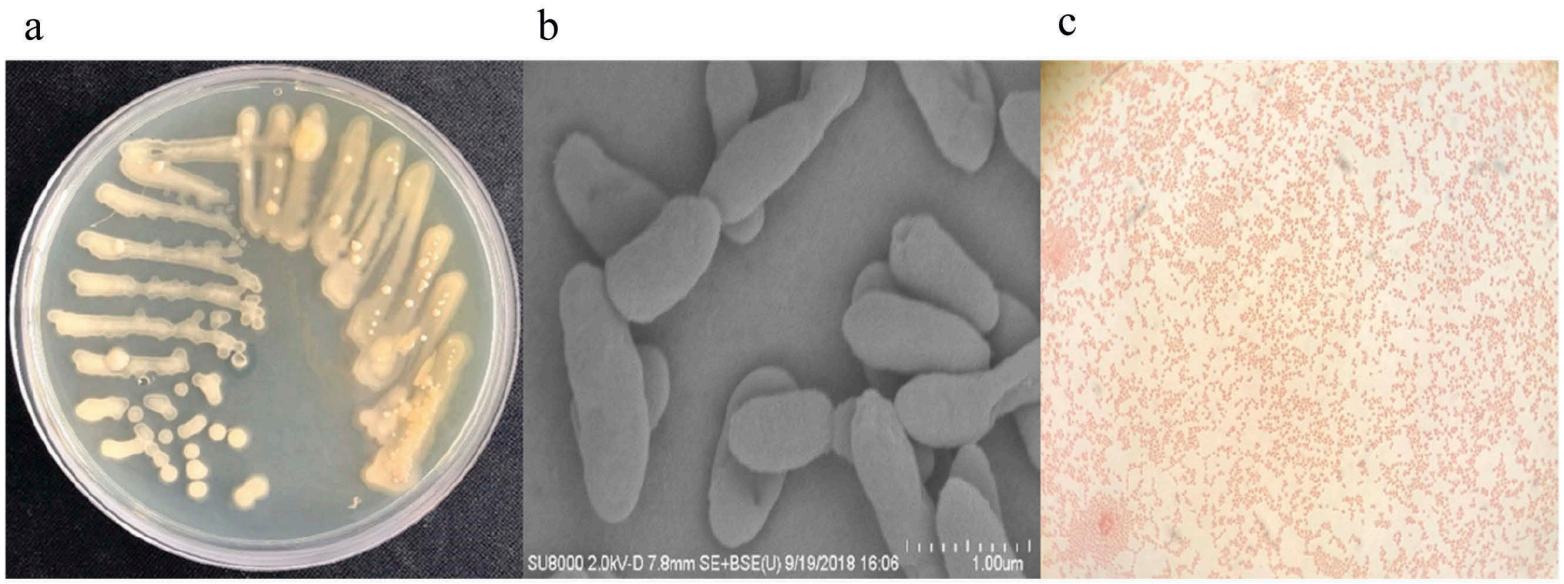
Strain streaking and purification plate, scanning electron microscopy and gram-staining. (a) Strain streaking and purification plate, forming a pale yellow and opaque colony size of 2–3.5mm. (b) Strain scanning electron microscopy, the specific morphology of the bacterial strain was observed by scanning electron microscopy. There was no obvious flagellum and the dimension ranges were 2.2–3.1 μm long, 0.4–0.9 μm wide. (c) The result of gram staining which was red under a light microscope was gram-negative bacteria.

*Pseudoalteromonas hodoensis* was obtained by experimental screening which produced gellan lyase with a high degradation ability. It formed obvious pits on PY medium, forming opaque, pale yellow round colonies with smooth edges, and moist surfaces with colony size of 2–3.5mm (Figure 3(a)). The bacteria were also without nuclei, spore formation or capsules, and with strictly anaerobic respiratory metabolism, with no fermentation. They were rod-shaped, with an average length of 2.3–3.1μm and diameter of 0.4–0.9μm (Figure 3(b)), and were highly efficient at gellan lyase production to reduce gellan molecular weight and viscosity. They were able to grow in a pH range of 5.5–8.5 at temperatures (20–50°C). The optimal conditions for their maximal growth were pH 7.0 and 30°C. The physiological and biochemical analyses results showed that the strain had no spores, flagella or capsules, and was gram-negative (Figure 3C). The arginine, ornithine, lysine, citrate and urea tests were positive, while the nitrate reduction, ONPG, sucrose, glucose, hydrogen sulfide, methyl red, MR-VP and indigo matrix tests were negative (Table 3).

### 3.4. Lyase activity detection

Gellan lyase (1.5 ml of crude enzyme) was added to 15 ml of 0.05% gellan solution in 50 mM Tris–HCl buﬀer with a pH of 7.0. After incubation at 30°C, the absorbance changes were monitored and recorded every 30 min in a Pye Unicam Spectrophotometer at 235 nm. An activity curve of lyase was then generated from the resulting data. The corresponding analytical curve showed that the light absorption value of the lyase strain at OD_235_ increased by 0.824U/ml/h within 24h, which was much stronger than the measured lyase activity of the previously reported *Geobacillus stearothermophilus* (0.03 U/ml/h) [15].

### 3.5. Optimization of the lyase conditions

**Figure 4.**
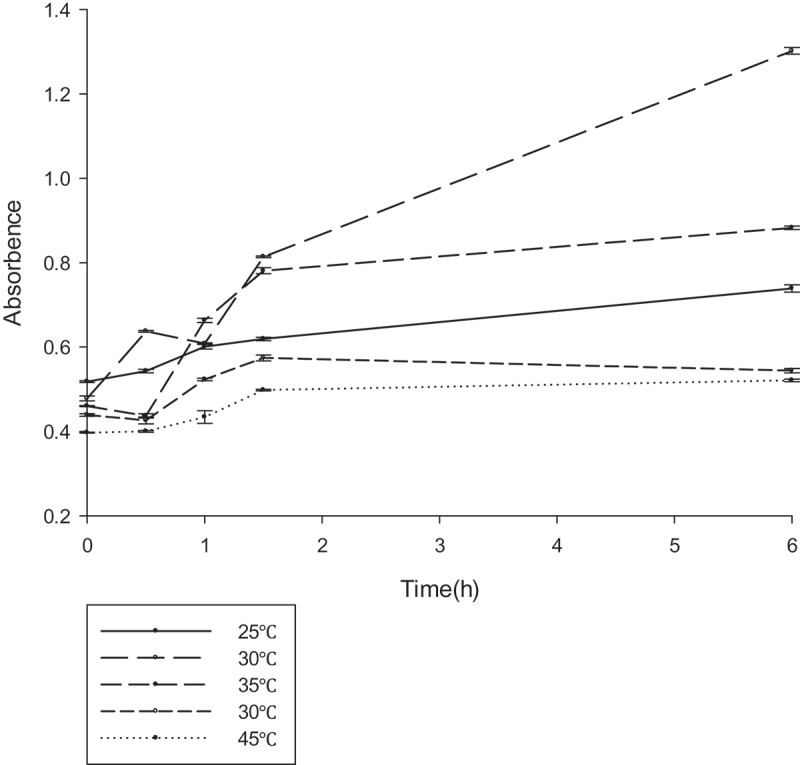
The optimum temperature reaction conditions for gellan lyase (unit:U/ml) .The enzyme was preincubated at pH 7.0 in 50 mM Tris–HCl buﬀer, with different temperature gradients. The lyase activity was determined by spectrophotometry at 235nm every 30 min.

**Figure 5.**
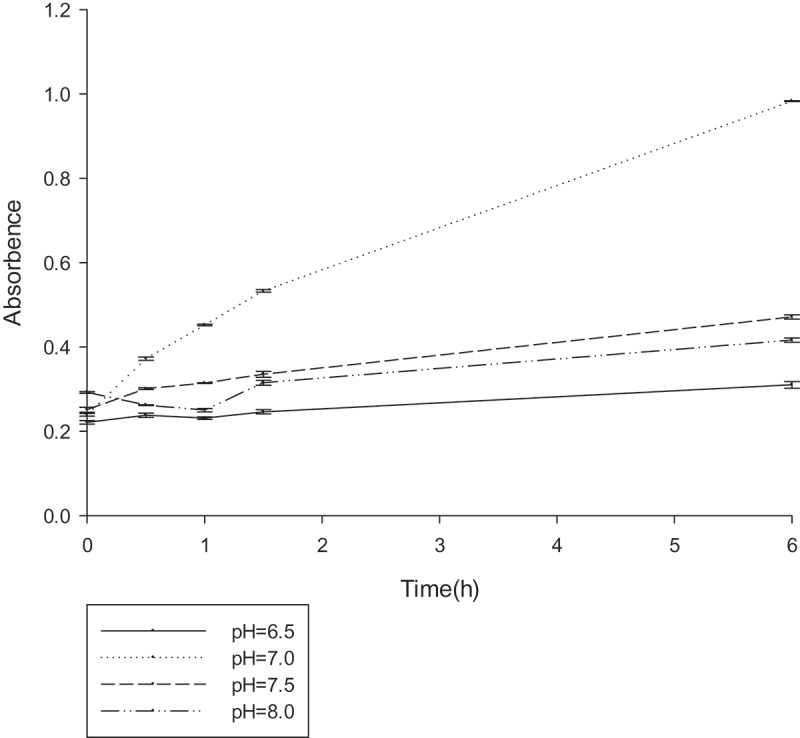
The optimum pH reaction conditions for gellan lyase (unit:U/ml).The enzyme was preincubated at 30°C in 50 mM Tris–HCl buﬀer, with different pH gradients. The lyase activity was determined by spectrophotometry at 235nm every 30 min.

To determine the optimal reaction conditions for gellan degradation, the effects of temperature (25–45°C), pH (6.5–7.5), and various compounds were examined. The gellan-degrading products were determined based on the accumulation of unsaturated glucuronyl ends. The absorbance at 235 nm was measured at different temperature ranges every 30 min (Figure 4), the lyase was active in the range of 25–45°C, with an optimum at 30°C. Similarly, the OD_235_ was measured at the different pH ranges every 30 min (Figure 5). From the results, it could be deduced that the lyase was active in the range of 6.5–7.5, with an optimum at 7.0. The effect of the various compounds on gellan lyase activity was also established (Table 1). In the presence of a number of metal ions in a low concentration (1 mM) a stimulating effect on the lyase activity was observed for almost all tested ions. Metal ions in high concentration (1 M) inhibited lyase activity. The increased activity in the presence of K^+^, NH_4_^+,^ and Na^+^ at such a concentration was no more than 10%. Mn^2+^ had the most promoting effect on PE1 lyase, by up to 138.39%. The lyase was sensitive to all the inhibitors, with the highest degree of inhibition towards urea.

**Table 3.** Physiological activity detection results.

Physiological activity detection	Results
Arginine	**+**
Ornithine	**+**
Lysine	**+**
Citrate	**+**
Urea	**+**
Nitrate reduction	-
ONPG	-
Sucrose	-
Glucose	-
Hydrogen sulfide	-
Methyl red	-
MR-VP	-
Indigo matrix	-

Biochemical identification tube for the physiological and biochemical detection of bacterial strains

**Table 1.** Effect of various ions and inhibitors on gellan lyase activity (unit:U/ml).

compound	0 min	60 min	difference value	relative activity（%
blank	0.212 *± 0.002*	0.324 *± 0.003*	0.112	100%
EDTA	0.242 *± 0.001*	0.327 *± 0.004*	0.085	75.89%
KCl	0.156 *± 0.001*	0.274 *± 0.005*	0.118	105.36%
MnCl_2_	0.221 *± 0.005*	0.376 *± 0.003*	0.155	138.39%
CaCl_2_	0.192 *± 0.001*	0.316 *± 0.001*	0.124	110.71%
NaCl	0.227 *± 0.003*	0.348 *± 0.003*	0.121	108.04%
(NH_4_)_2_SO_4_	0.415 *± 0.004*	0.532 *± 0.002*	0.117	104.46%
urea	0.164 *± 0.002*	0.199 *± 0.003*	0.048	42.86%

The enzyme was preincubated for 30 min at 30°C in Tris–HCl buffer, pH 7.0, containing different ions and inhibitors at final concentration 1 mM and the activity was then determined at 30°C, pH 7.0. The difference value was calculated by the difference between absorbance at the initial time 0 min and reaction time at 60 min. At the same time, relative activity was determined by comparing the difference between different ions or compounds and the blank control.

### 3.6. Thin-layer chromatography and high-performance liquid chromatography

**Figure 6.**
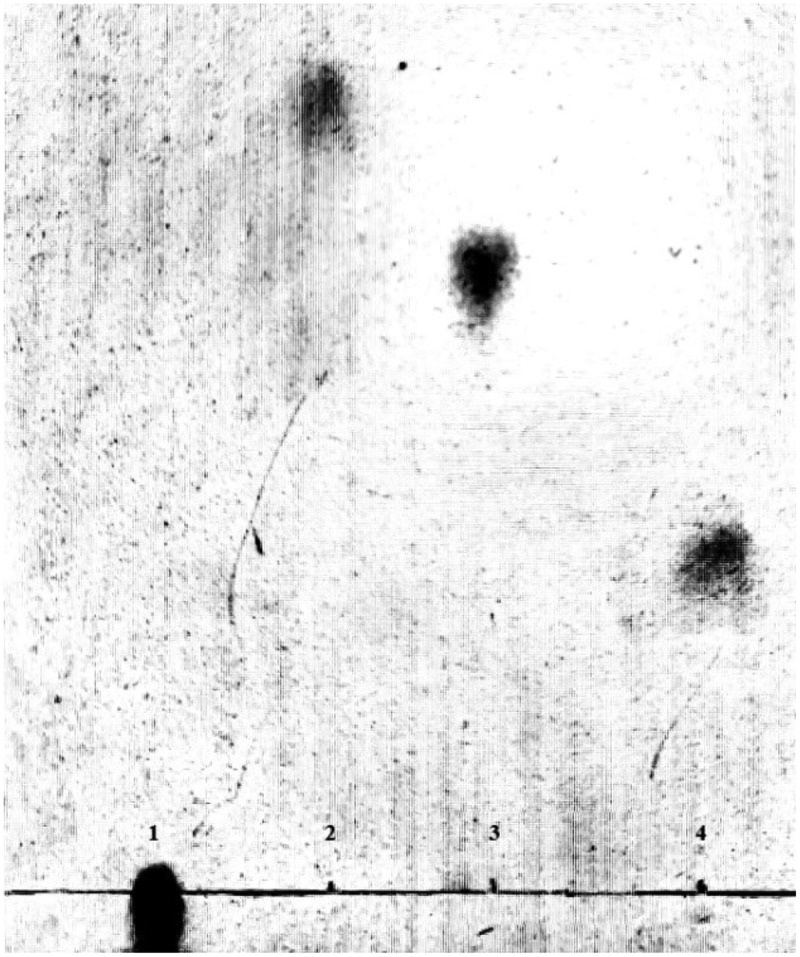
TLC profile of the gellan degradation product after 48h incubation. Lane 1, gellan (0.05%); lane 2, rhamnose (1%); lane 3, glucose (1%); lane 4, gellan degradation product after 48h incubation.

**Figure 7.**
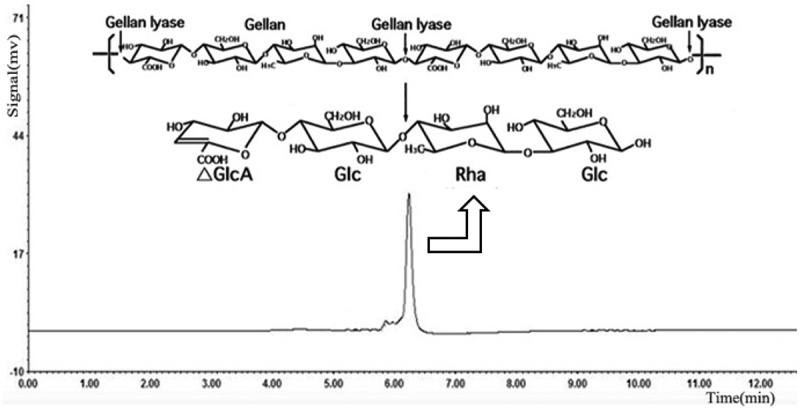
HPLC analysis of the gellan degradation products. Gellan was converted to a tetrasaccharide (4,5-ene-glucuronyl-glucosyl-rhamnosyl-glucose) by the gellan lyase.

Under the optimum culturing conditions determined above, lyase degraded gellan efficiently and the culture supernatant showed single spots on the TLC plates, which was thought to be an oligosaccharide product. On a TLC plate, spots of gellan-degraded products were distinguished from the components of gellan polymer (Figure 6). Gellan is formed by linear tetrasaccharide repeating units, hence its rise in the TLC plate was extremely slow, but rhamnose and glucose, being monosaccharides, had very fast ascending development. On the TLC plate, the spots of gellan-degrading products were lower than those of rhamnose and glucose, but higher than gellan. This indicated that the molecular structure of gellan was degraded by the action of the lyase. When the reaction products of lyase were analyzed by HPLC. Results displayed that the purified oligosaccharides samples had a single absorption peak at 235nm. Compared with the glucan standard substance in the same condition, we confirmed almost all the degradation products were the minimum enzymatic degradation products(tetrasaccharide units containing double bond) (Figure 7) [29]. Therefore, we speculate that the lyase through β-elimination broke down the 1,4-glycosidic bonds associated with the β-D-glucuronic acid, formed unsaturated oligosaccharides. It was consistent with the degradation mechanism of acidic polysaccharides.

## 4. Discussion

Although gellan is of great interest for many commercial applications, the expensive downstream processing impairs the economic viability of gellan production, and consequently, it is the most expensive among the various food gums [30]. Additionally, its highly viscous properties have limited its utility, particularly in the food industry [31]. Therefore, we chose to use efficient gellan lyase to improve its molecular structure and expand its application range.

Emphatically, the newly isolated strain of *Pseudoalteromonas hodoensis* is the ﬁrst reported gellan lyase producing bacteria in marine *Pseudoalteromonas*. It has very high efficiency in degrading gellan gum compared with the bacteria producing gellan lyase obtained in previous studies, such as in the work by Derekova et al (2006) on *Geobacillus stearothermophilus*(crude lyase 0.03U/ml/h vs 0.824U/ml/h) [15]. At the same time, the optimum action conditions of lyase were 30°C and pH 7.0, so environmental impacts are mild. And the oligosaccharide molecular weight produced by gellan lyase was single, this indicates that the lyase can degrade gellan specifically and the purification cost is very low. Furthermore, through bio-enzymatic degradation, its reaction is controllable with low energy consumption and high lyase yield [32]. By comparison, it has great advantages over the existing industrial degradation methods. Therefore, it has very important applications in fermentation processes as well as in biomass energy utilization [33].

The application of degradation products in new fields will provide a new direction for the market application of polysaccharides [34]. Such as Salina et al. have found that gellan oligosaccharides can promote the growth and antibacterial activity of *Eucommia ulmoides* [35]. Polysaccharide lyases are believed to provide tremendous commercial beneﬁts, but their production at industrial levels still remains a challenge. At present, the lack of suitable high activity lyase is the biggest obstacle for its application. The ideal lyase should be characterized by high activity, mild operating conditions, and a single product. Therefore, we believe that lyase from *Pseudoalteromonas hodoensis* met these criteria and is suitable for a wide range of industrial applications.

Through preliminary detection, we can conclude that the lyase obtained from *Pseudoalteromonas hodoensis* has strong ability to degrade gellan. In addition, the preliminary analysis of the active site of lyase has also been completed. As part of our future works, we will be purifying and collecting lyase to detect its yield, and the whole genome of the strain will be sequenced to analyze the relevant genes associated with lyase production. This is a preliminary exploration on our part to obtain lyase in large quantities for subsequent oligosaccharide preparation and industrial application. A promising way might be cloning the genes, and this is also part of our follow-up research. We would also like to continue developing the application fields of gellan oligosaccharides by detecting the antibacterial, antioxidant and intestinal probiotics activities of the oligosaccharides. The application of polysaccharide derivatives has been greatly enhanced by the broadening research on polysaccharide lyases and their products.

## References

[1] Danalache F, Beirão-Da-Costa S, Mata P, et al Texture, microstructure and consumer preference of mango bars jellified with gellan gum. LWT-Food Sci Technol. 2015;62:584–591.

[2] Lin KW, Huang H. Konjac/gellan gum mixed gels improve the quality of reduced-fat frankfurters. Meat Sci. 2003;65:749–755. 22063436 10.1016/S0309-1740(02)00277-2

[3] Pacelli S, Paolicelli P, Avitabile M, et al Design of a tunable nanocomposite double network hydrogel based on gellan gum for drug delivery applications. Eur Polym J. 2018;104:104–183.

[4] Danalache F, Mata P, Moldão-Martins M, et al Novel mango bars using gellan gum as gelling agent: rheological and microstructural studies. LWT-Food Sci Technol. 2015;62:576–583.

[5] Yip V, Withers S Nature’s many mechanisms for the degradation of oligosaccharides. Org Biomol Chem. 2004;2:2707–2713. 15455137 10.1039/B408880H

[6] Garron M, Cygler M Structural and mechanistic classification of uronic acid-containing polysaccharide lyases. Glycobiology. 2010;20:1547–1573. 20805221 10.1093/glycob/cwq122

[7] Gacesa P Alginate-modifying enzymes: A proposed unified mechanism of action for the lyases and epimerases. Febs Lett. 1987;212:199–202.

[8] Jansson P, Lindberg B, Sandford P Structural studies of gellan gum, an extracellular polysaccharide elaborated by *Pseudomonas elodea*. Carbohyd Res. 1983;124:135–139.

[9] Mikolajczak M, Thorne L, Pollock TJ, et al Sphinganase, a new endoglycanase that cleaves specific members of the gellan family of polysaccharides. Appl Environ Microb. 1994;60:402. 10.1128/aem.60.2.402-407.1994 PMC201327 8135511

[10] Mcguffey J, Leon D, Dhanji E, et al Bacterial production of gellan gum as a Do-It-Yourself alternative to Agar. J Microbiol Bio Edu. 2018;19:1–3. 10.1128/jmbe.v19i2.1530 PMC6022777 29983852

[11] Elboutachfaiti R, Delattre C, Petit E, et al Polyglucuronic acids: structures, functions and degrading enzymes. Carbohyd Polym. 2011;84:1–13.

[12] Douglas T, Pilarz M, Lopez-Heredia M, et al Composites of gellan gum hydrogel enzymatically mineralized with calcium-zinc phosphate for bone regeneration with antibacterial activity. J Regen Med Tissue Eng. 2017;102:129–138. 10.1002/term.2062 26174042

[13] Redouan E, Emmanuel P, Michelle P, et al Evaluation of antioxidant capacity of ulvan-like polymer obtained by regioselective oxidation of gellan exopolysaccharide. Food Chem. 2011;127:976–983. 25214086 10.1016/j.foodchem.2011.01.067

[14] Tetsuguchi M, Nomura S, Katayama M, et al Effects of curdlan and gellan gum on the surface structure of intestinal mucosa in rats. J Nutr Sci Vitaminol. 1997;43:515–527. 9505237 10.3177/jnsv.43.515

[15] Derekova A, Sjøholm C, Mandeva R, et al Biosynthesis of a thermostable gellan lyase by newly isolated and characterized strain of *Geobacillus stearothermophilus 98*. Extremophiles. 2006;10:321–326. 16482399 10.1007/s00792-005-0503-y

[16] Hashimoto W, Momma K, Miki H, et al Enzymatic and genetic bases on assimilation, depolymerization, and transport of heteropolysaccharides in bacteria. J Biosci Bioeng. 1995;87:123–136. 10.1016/s1389-1723(99)89001-x 16232439

[17] Peng C, Wang Q, Wang S, et al A chondroitin sulfate and hyaluronic acid lyase with poor activity to glucuronyl 4,6-O-disulfated N-acetylgalactosamine (E-type)-containing structures. J Biol Chem. 2018;2934230–4243. 10.1074/jbc.RA117.001238 PMC5868263 29414785

[18] Cruz A, Ishii T Arbuscular mycorrhizal fungal spores host bacteria that affect nutrient biodynamics and biocontrol of soil-borne plant pathogens. Biol Open. 2012;1:52–57. 23213368 10.1242/bio.2011014 PMC3507164

[19] Ullah H, Badshah M, Mäkilä E, et al Fabrication, characterization and evaluation of bacterial cellulose-based capsule shells for oral drug delivery. Cellulose. 2017;24:1445–1454.

[20] Fujii T, Matsunami H, Inoue Y, et al Evidence for the hook supercoiling mechanism of the bacterial flagellum. Biophys Physicobiol. 2018;15:28–32. 29607277 10.2142/biophysico.15.0_28 PMC5873038

[21] Mayfield C, Inniss W A rapid, simple method for staining bacterial flagella. Can J Microbiol. 1977;23:1311–1313. 71191 10.1139/m77-198

[22] Snm R, Milani E, Deed R, et al Bacteria, mold and yeast spore inactivation studies by scanning electron microscope observations. Int J Food Microbiol. 2017;263:17–25. 29024903 10.1016/j.ijfoodmicro.2017.10.008

[23] Videira P, Fialho A, Geremia RA Biochemical characterization of the β-1,4-glucuronosyltransferase GelK in the gellan gum-producing strain S*phingomonas paucimobilis* ATCC 31461[J]. Biochem J. 2001;358:457–464. 11513745 10.1042/0264-6021:3580457 PMC1222079

[24] Hashimoto W, Inose T, Nakajima H Purification and characterization of microbial gellan lyase.[J]. Appl Environ Mibrob. 1996;62:1475–1477. 10.1128/aem.62.4.1475-1477.1996 PMC167921 8919816

[25] Hashimoto W, Murata K Alpha-L-rhamnosidase of *Sphingomonas sp*. R1 producing an unusual exopolysaccharide of sphingan. Biosci Biotechnol Biochem. 1998;62:1068–1074. 9692187 10.1271/bbb.62.1068

[26] Chen C, Cong YY, Remila M Analysis of monosaccharide compositions of 2 kinds of Pleurotus polysaccharides by thin layer chromatography and gas chromatography. J Biosci Bioeng. 2017;10:345–367.

[27] Yan J, Guo XQ, Xiao-Guang LI, et al TLC to Fleetly analyze monosaccharide composition of polysaccharide. Food Sci. 2006;27:603–605.

[28] Huang C, Gao XD, Chen HJ, et al A simple method to analyze monosaccharide composition of polysaccharide. Shangdong Pharm Ind. 2005;5:24–29.

[29] Jansson P, Lindberg B, Linberg J, et al Structural studies of a polysaccharide (S-194) elaborated by *Alcaligenes* ATCC 31961. Carbohydr Res. 1986;156:157–163. 10.1016/s0008-6215(00)90108-3 3815405

[30] Kohajdová Z, Karovičová J Application of hydrocolloids as baking improvers. Chem Papers. 2009;63:26–38.

[31] Gal A, Nussinovitch A Hydrocolloid carriers with filler inclusion for diltiazem hydrochloride release. J Pharm Sci. 2010;96:168–178. 10.1002/jps.20775 17031844

[32] Miyake O, Kobayashi E, Nankai H, et al Posttranslational processing of polysaccharide lyase: maturation route for gellan lyase in *Bacillus sp*. GL1 Arch Biochem Biophys. 2004;422:211–220. 14759609 10.1016/j.abb.2003.12.015

[33] Donot F, Fontana A, Baccou J, et al Microbial exopolysaccharides: main examples of synthesis, excretion, genetics, and extraction. Carbohyd Polym. 2012;87:951–962.

[34] Sutherland I Polysaccharides for microbial exopolysaccharides. Carbohyd Polym. 1999;38:319–328.

[35] Salachna P Mizielińska, M., Soból, M. Exopolysaccharide gellan gum and derived oligo-gellan enhance growth and antimicrobial activity in Eucomis plants. Polymers. 2018;10:242–253. 10.3390/polym10030242 PMC6414989 30966277

